# Efficacy of a Trivalent Hand, Foot, and Mouth Disease Vaccine against Enterovirus 71 and Coxsackieviruses A16 and A6 in Mice

**DOI:** 10.3390/v7112916

**Published:** 2015-11-17

**Authors:** Elizabeth A. Caine, Jeremy Fuchs, Subash C. Das, Charalambos D. Partidos, Jorge E. Osorio

**Affiliations:** 1 Department of Pathobiological Sciences, School of Veterinary Medicine, University of Wisconsin-Madison, Madison, WI 53706, USA; jorge.osorio@wisc.edu; 2 Takeda Vaccines Inc., Madison, WI 53719, USA; jeremy.fuchs@takeda.com (J.F.); subash.das@takeda.com (S.C.D.); harry.partidos@takeda.com (C.D.P.)

**Keywords:** Enterovirus 71, Coxsackievirus A16, Coxsackievirus A6, AG129 mice, mouse model, trivalent vaccine, interferon

## Abstract

Hand, foot, and mouth disease (HFMD) has recently emerged as a major public health concern across the Asian-Pacific region. Enterovirus 71 (EV71) and Coxsackievirus A16 (CVA16) are the primary causative agents of HFMD, but other members of the *Enterovirus A* species, including Coxsackievirus A6 (CVA6), can cause disease. The lack of small animal models for these viruses have hampered the development of a licensed HFMD vaccine or antivirals. We have previously reported on the development of a mouse model for EV71 and demonstrated the protective efficacy of an inactivated EV71 vaccine candidate. Here, mouse-adapted strains of CVA16 and CVA6 were produced by sequential passage of the viruses through mice deficient in interferon (IFN) α/β (A129) and α/β and γ (AG129) receptors. Adapted viruses were capable of infecting 3 week-old A129 (CVA6) and 12 week-old AG129 (CVA16) mice. Accordingly, these models were used in active and passive immunization studies to test the efficacy of a trivalent vaccine candidate containing inactivated EV71, CVA16, and CVA6. Full protection from lethal challenge against EV71 and CVA16 was observed in trivalent vaccinated groups. In contrast, monovalent vaccinated groups with non-homologous challenges failed to cross protect. Protection from CVA6 challenge was accomplished through a passive transfer study involving serum raised against the trivalent vaccine. These animal models will be useful for future studies on HFMD related pathogenesis and the efficacy of vaccine candidates.

## 1. Introduction

In recent decades, many members of the species *Enterovirus A* have re-emerged causing increased incidence of hand, foot, and mouth disease (HFMD) across the Asian-Pacific region [1]. The disease mostly affects young children and is characterized by ulcers on the hands, feet, and oral cavity of infected individuals [1]. In some instances, neurological manifestations are observed including aseptic meningitis, brainstem encephalitis, pulmonary edema, and polio-like paralysis [1]. HFMD is primarily caused by Enterovirus 71 (EV71; *Picornaviridae*; *Enterovirus*) and Coxsackievirus A16 (CVA16; *Picornaviridae*; *Enterovirus*), but occasionally other non-polio enteroviruses, including Coxsackievirus A6 (CVA6; *Picornaviridae*; *Enterovirus*), are involved [2]. The disease is now endemic in most Asian-Pacific countries, but serious outbreaks still occur. For example, China had 1.7 million HFMD cases in 2010 and Vietnam reported 113,121 children infected with 170 deaths in 2011 [3,4]. Japan, Korea, Singapore, and Cambodia also have suffered from the recent re-emergence of HFMD [5,6,7], and cases also have increased in other parts of the world including India, Cuba, Europe, and the United States [8,9,10]. As a result, vaccination has been considered a necessary component for HFMD prevention and control; however, vaccination against HFMD is challenging. For example, there are many different enteroviruses that cause HFMD with no cross protection between them, making a universal vaccine difficult to formulate [11,12]. There is also a lack of adult, small animal models to human enteroviruses preventing the evaluation of vaccine efficacy and safety.

Early HFMD vaccine efforts began with an inactivated form of EV71. Sinovac (Beijing, China), CAMS (Beijing, China), and Beijing Vigoo (Beijing, China), all completed phase 1, 2, and 3 clinical trials with an inactivated C4 sub-genogroup EV71 vaccine [13,14,15]. It has also been shown that immune sera from immunized individuals cross-neutralize other circulating EV71 genogroups, but fails against other HFMD enteroviruses [16]. Inactivated CVA16 vaccines and virus-like particles have also been shown to produce strong neutralizing antibody responses against homologous and heterologous viruses and protected against challenge in neonatal mice [17]. Experimental bivalent EV71:CVA16 vaccines elicited protective responses against both viruses, however testing of these vaccines is still needed to characterize protection against other HFMD enteroviruses [11,18].

Accordingly, we present our work on: (1) the development of mouse models for CVA16 and CVA6; and (2) the use of these models plus our previously developed model for EV71 to test the efficacy of an inactivated trivalent (EV71:CVA16:CVA6) vaccine. This work demonstrates the importance of having a small animal model to test candidate vaccines and also documents the success of a trivalent vaccine in providing protection to three HFMD causing viruses. Future studies will be designed to use these models to analyze the pathogenesis and map virulence domains for viruses belonging to the *Enterovirus A* species.

## 2. Results

### 2.1. Mouse Adaptation of CVA16 and CVA6

**Figure 1:**
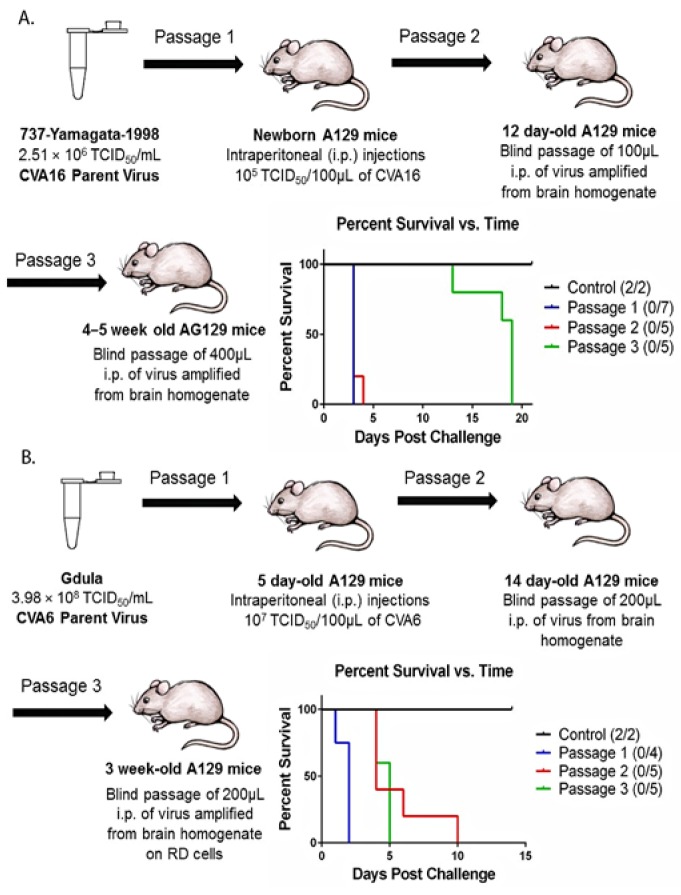
Timeline for mouse adaptation of CVA16 and CVA6. (**A**) CVA16 viral strain 737-Yamagata-1998 was passed three times in young A129 and AG129 mice, increasing age with each passage. Intraperitoneal (i.p.) injections were done for all viral challenges, with negative control groups receiving 1× PBS (same route and volume). All mice challenged with virus developed clinical signs of disease and succumbed to infection; (**B**) CVA6 viral strain Gdula was passed three times in young A129 mice using the same methodology described above. All mice succumbed to infection in each passage, with clinical signs developing after 10 days for the final passage.

Blind serial passages of CVA16 resulted in the production of an adapted virus that was lethal in 12-week-old AG129 (α/β and γ interferon (IFN) receptor deficient) mice at a dose of 9.8 × 10^5^ TCID_50_ units in 400 µL. During the first two passages of CVA16, all mice developed clinical signs of disease and succumbed to infection by day 3. Clinical signs included weight loss, hunched posture, limb paralysis, eye inflammation, incoordination, and death. Interestingly, generation of P3 virus resulted in a delayed onset of clinical symptoms (day 12) with 100% mortality by day 19 (Figure 1). CVA16-P3 was used to challenge 12 week-old AG129 mice, where clinical signs developed by day 12, and 100% of mice succumbed to infection by day 18.

Similar results were observed during adaptation of CVA6 in newborn to 3 week-old A129 (α/β IFN receptor deficient)   mice. 3 week-old mice injected with P3 virus developed clinical signs (day 3) and succumbed to infection on day 5 (Figure 1). Surprisingly, when CVA6-P3 virus was used to challenge 12-week-old AG129 mice, they did not develop clinical signs or succumb to infection. This indicated that passage of CVA6 in 12 week-old interferon deficient mice was not productive. Another passage was done in 4 week-old AG129 mice, but adaptation was still unsuccessful. It is unclear why the mouse adaptation methodology worked with three passages for our previously developed EV71 model and CVA16, but not CVA6.

**Figure 2:**
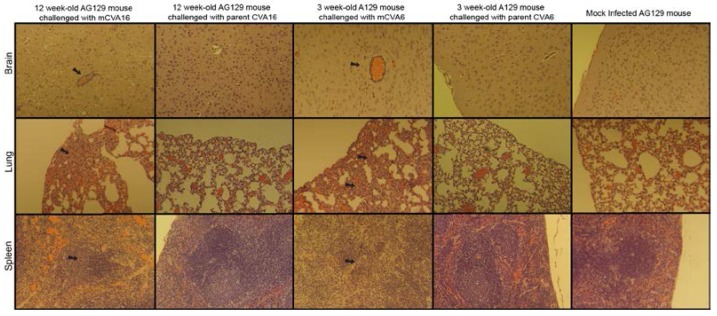
Histopathological changes associated with interferon deficient mice challenged with mouse adapted CVA16 and CVA6. Pictures of tissues were done with a 10× objective. First column shows tissues from a 12 week-old AG129 mouse challenged with mCVA16. The third, tissues from a 3 week-old A129 mouse challenged with mCVA6. Perivascular cuffing is observed in the brain, interstitial pneumonia in the lungs, and lymphoid depletion in the spleen of mice infected with both mouse adapted viruses (see black arrows). The last column displays tissues from a mock infected mouse. Mice challenged with the parent strains of each virus did not show clinical signs of disease or pathology in any tissues.

Tissue samples from 12 week-old AG129 mice challenged with mCVA16 or 3 week-old A129 mice challenged with mCVA6 displayed perivascular cuffing in the brain, lymphoid depletion in the spleen and interstitial pneumonia in the lungs (Figure 2). Mice challenged with the parent viruses did not show clinical signs of disease and tissue samples did not have any significant histological changes.

### 2.2. Efficacy of HFMD Vaccines against mEV71 Challenge

**Figure 3:**
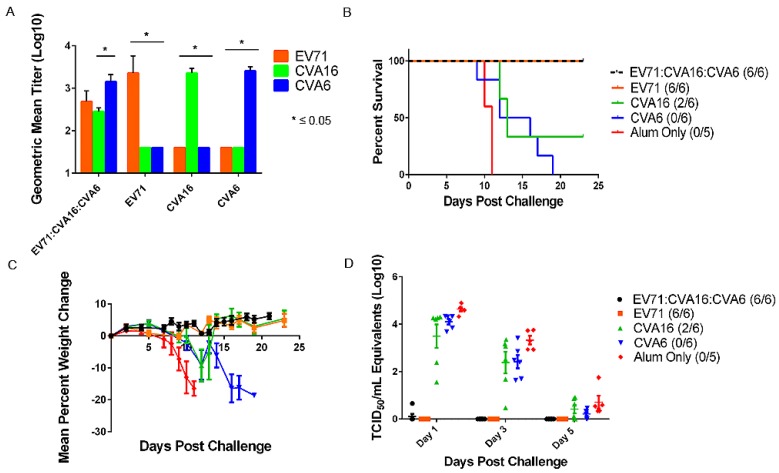
Monovalent EV71 and trivalent EV71:CVA16:CVA6 vaccines provided complete protection to challenge with mEV71 in 10 week old AG129 mice. (**A**) Microneutralization assays were performed on serum collected on day 14 post boost against EV71, CVA16, and CVA6. Each monovalent vaccine had relatively high levels of neutralizing antibodies to its homologous strain, but showed no cross protection against other viruses. The lack of protection by monovalent vaccine against other viruses is presented in panel B. The trivalent vaccinated mice developed neutralizing antibodies to all three viruses with a significant higher level of CVA6 neutralizing antibodies compared to CVA16; (**B**) The monovalent EV71 and trivalent vaccines elicited complete protection against lethal challenge with mEV71. Alum only and CVA6 vaccinated groups succumbed to infection, while 2 out of 6 mice survived in the monovalent CVA16 vaccinated group; (**C**) Weight loss is a definitive clinical sign in mice challenged with mEV71. Monovalent CVA6 vaccinated group and alum only both develop significant weight losses. The weight gain seen after day 13 post challenge in the monovalent CVA16 vaccinated group is due to the two surviving mice; (**D**) EV71 viral loads were measured by real time RT-PCR. Significant viral loads were detected on days 1 and 3 in the monovalent CVA16, CVA6, and alum only groups. No detectable viral loads were detected in the monovalent EV71 and trivalent vaccinated groups.

To evaluate the protective efficacy of an inactivated trivalent vaccine candidate against mEV71 challenge, an active immunization study was conducted in AG129 mice. Mice were vaccinated as described with either a monovalent (EV71, CVA16, or CVA6) or a trivalent vaccine and challenged with our previously adapted mEV71. On day 14 post boost, the trivalent vaccine induced high neutralizing antibody titers against all three viruses with GMTs of 485 for EV71, 285 for CVA16, and 1436 for CVA6 (Figure 3). Mice that received monovalent vaccines had high levels of neutralizing antibodies to their homologous virus with no detectable levels of cross neutralizing antibodies to the other viruses (CVA16 and CVA6). Interestingly, significantly lower levels of CVA16 antibodies were detected in the trivalent formulation when compared to the monovalent CVA16 (GMT = 2280) (*p* ≤ 0.05). Monovalent EV71 and trivalent groups were protected 100% from lethal mEV71 challenge (Figure 3). This was statistically significant when compared to alum only control (*p* < 0.05).

All mice vaccinated with monovalent CVA6 and challenged with mEV71 developed clinical signs of disease, viral loads, and succumbed to infection (Figure 3). Two animals from the CVA16 vaccinated group survived challenge and developed lower viral loads, however 4/6 animals still succumbed to infection showing a lack of cross protection from the vaccine. Significant weight loss was observed on days 12 and 13 for the CVA16 vaccinated groups, past day 12 for CVA6 vaccinated groups, and past day 8 for the alum only group. There were no detectable EV71 viral loads in the trivalent and monovalent EV71 vaccine groups; whereas, statistically significant levels (*p* < 0.05) were seen in the alum only, and monovalent CVA16 and CVA6 groups on days 1 and 3 (Figure 3).

**Figure 4:**
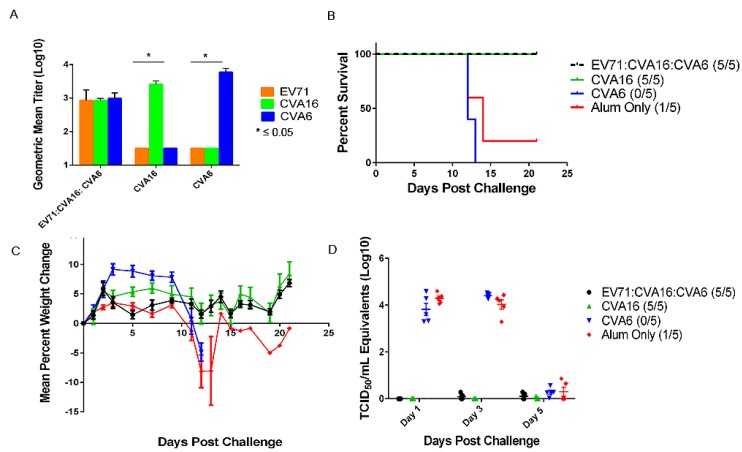
Monovalent CVA16 and trivalent EV71:CVA16:CVA6 vaccines provided complete protection to challenge with mCVA16 in 10 week-old AG129 mice. (**A**) Microneutralization assays were performed on serum collected on day 14 post boost against EV71, CVA16, and CVA6. Each monovalent vaccine had relatively high levels of neutralizing antibodies to its homologous strain, but showed no cross protection against other viruses. The lack of protection by the CVA6 monovalent vaccine against CVA16 is presented in panel B. The trivalent vaccinated mice developed neutralizing antibodies to all three viruses; (**B**) The monovalent CVA16 and trivalent vaccines elicited complete protection against lethal challenge with mCVA16. Alum only and CVA6 vaccinated groups had a significant number of animals succumbed to infection; (**C**) Weight loss is a definitive clinical sign in mice challenged with mCVA16. Monovalent CVA6 vaccinated group and alum only both developed significant weight losses. The weight gain seen after day 13 post challenge in the alum only vaccinated group is due to one surviving mouse; (**D**) CVA16 viral loads were measured by real time RT-PCR. Significant viral loads were detected on days 1 and 3 in the monovalent CVA6, and alum only groups. No detectable viral loads were detected in the monovalent CVA16 and trivalent vaccinated groups. All mice in the alum only group had detectable viral loads, even the one mouse that survived challenge.

### 2.3. Efficacy of HFMD Vaccines against mCVA16 Challenge

An active immunization study was also conducted to evaluate the protective efficacy of the trivalent and monovalent HFMD vaccines against mCVA16 challenge. Mice receiving monovalent vaccines had high levels of neutralizing antibodies to their homologous virus with no detectable levels of cross neutralizing antibodies to the others (Figure 4). Mice receiving the trivalent vaccine developed neutralizing antibodies to all three viruses (GMT = 844-EV71, 844-CVA16, 970-CVA6) with significantly lower levels of CVA16 and CVA6 antibodies when compared to the monovalent CVA16 (GMT = 3378) and CVA6 (GMT = 5881) (*p* ≤ 0.05). Monovalent CVA16 and trivalent groups were protected 100% from lethal mCVA16 challenge (Figure 4). This was statistically significant when compared to alum only control mice (*p* < 0.05). Vaccination with monovalent vaccines did not provide protection against heterologous challenge. For example, mice vaccinated with monovalent CVA6 and challenged with mCVA16 developed clinical signs of disease, viral loads in serum, and succumbed to infection (Figure 4). Significant weight loss was seen in challenged groups on days 11–13 for the alum only group and days 5, 7, 9, 12 for CVA6 vaccinated groups. Trivalent and CVA16 vaccinated groups showed no detectable levels of virus, but a statistically significant level was seen for both the alum only and monovalent CVA6 vaccinated groups on days 1 and 3 (*p* ≤ 0.05).

### 2.4. Efficacy of HFMD Vaccines against mCVA6 Challenge

**Figure 5:**
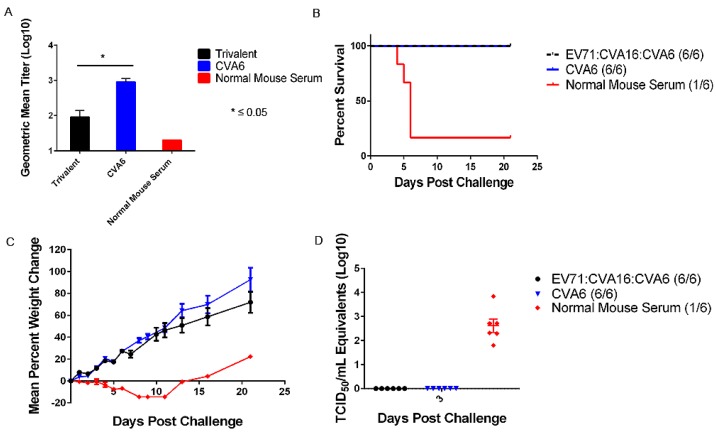
Monovalent CVA6 and trivalent antibodies provided protection against lethal challenge with mCVA6 in 3 week-old A129 mice. (**A**) Microneutralization assays were performed on serum collected 24 h post transfer to test for circulating antibody titers to CVA6. A significant difference was seen between the circulating titers in the group receiving monovalent CVA6 antibodies compared to the trivalent group; (**B**) The monovalent CVA6 and trivalent antibodies elicited complete protection against lethal challenge with mCVA6. A significant number of animals receiving normal mouse serum succumbed to infection; (**C**) Weight loss is a definitive clinical sign in mice challenged with mCVA6. Mice receiving normal mouse serum developed significant weight losses. The weight gain seen after day 11 post challenge in the normal mouse serum group is due to the one surviving mouse; (**D**) CVA6 viral loads were measured by real time RT-PCR. Significant viral loads were detected on day 3 in the normal mouse serum group. No detectable viral loads were detected in the CVA6 and trivalent groups.

To determine whether antibodies present in AG129 vaccinated mice were sufficient to provide protection against CVA6, pooled immune serum raised during active vaccination studies was passively transferred into 3 week-old AG129 mice. As a positive control, a group of mice received serum from naïve AG129 mice. The levels of circulating antibodies 24 h after injection were moderate in mice receiving the trivalent formulation (GMT = 140) and the monovalent CVA6 (GMT = 1013) (Figure 5). All mice treated with anti-CVA6-monovalent or anti-trivalent immune serum were protected 100% from lethal mCVA6 challenge (Figure 5). A significant amount of mice (4/5) passively transferred with serum from naïve mice developed clinical signs of disease, significant viral loads, and succumbed to infection (Figure 5).

## 3. Discussion

The increased incidence of HFMD related enterovirus infections, including EV71, CVA16, and CVA6, has led to a search for a safe and broadly neutralizing vaccine. However, development of a HFMD vaccine has been hampered by the lack of a small animal model and the inability of these viruses to produce cross neutralizing antibodies. Here, mouse models were developed that facilitated comparisons of efficacy between inactivated monovalent and trivalent vaccines. These models provide useful information on the cross neutralizing potential of these viruses during *in vivo* challenge. The efficacy of a candidate trivalent HFMD vaccine was shown to broadly protect mice against EV71 and CVA16 challenge. Furthermore, passive transfer studies with CVA6 demonstrated the importance of neutralizing antibodies in controlling HFMD-related enterovirus infections.

Humans are the only known natural host of enteroviruses. Nevertheless, there have been numerous attempts to develop small animal models for *in vivo* challenge studies using these viruses [19,20,21], with a primary focus on newborn immunocompetent mice and EV71 [21]. Strategies have included adaptation [22], testing in a variety of animals [21,23], as well as the development of a transgenic mouse model [24]; however, all of these models fail to increase the age of susceptibility to EV71 beyond three weeks of age. The dependence on young animals limits the applicability of these models for efficacy testing of potential vaccine candidates. Furthermore, animal models for CVA16 and CVA6 are even less developed [17,25,26]. Only neonatal mouse models have been characterized for these viruses.

Previously, we demonstrated that the combined use of IFN deficient mice and adaptation can increase the age of EV71 susceptibility to 12 week-old mice. Despite the lack of a functional IFN response, A129 and AG129 mice do develop humoral and cellular T-cell responses [27,28,29]. Furthermore, the usefulness of IFN deficient mice in the development of animal models has been demonstrated in numerous studies [21,30,31]. We also used our EV71 model to successfully test the efficacy of a monovalent EV71 vaccine that successfully completed phase I clinical trials [32,33]. Using similar methodology, we were able to produce a mouse adapted strain for CVA16. To our knowledge this is the first mouse model other than a neonatal model for CVA16 [26]. The adaptation of CVA6 was less successful with the mouse adapted virus only producing clinical signs in 15 day-old A129 mice. CVA6 causes an atypical HFMD reaction in which lesions are often generalized and more widely distributed, including dorsal sides of hands and feet, calves and trunk [34,35] It also uses unidentified cellular receptors to infect RD cells that differ from the receptors used by CVA16 and EV71 [36,37,38]. The difference in natural infection between these viruses may explain the lack of adaptation. Even with further passaging in younger 3 week-old A129 and AG129 mice, susceptibility of mice to CVA6 never surpassed 3 weeks. We are currently exploring the factors responsible for limiting CVA6 adaptation.

The clinical signs we observe in our mouse models (paralysis, neurological complications) mimic what is seen in severe enterovirus infections in humans. EV71 infections resulted in 3,046 deaths in China between 2008 and June 2014 [39]. Signs of brain encephalitis (perivascular cuffing) was previously reported in mice infected with our mouse adapted EV71 and newly mouse adapted CVA16. Interestingly, something not seen with our EV71 model, CVA16 and CVA6 infection caused interstitial pneumonia in the lungs that compares to pulmonary edema seen in natural human infection. Drawbacks to our model include the fact that neurological signs were observed with all of the viruses, which is not characteristic of human infection, and most likely the result of extracting virus from the brain during adaptation. The lack of IFN also allows the virus to spread systemically with dramatic clinical signs. Future studies will be necessary to further analyze pathogenesis associated with these models.

Many candidate inactivated monovalent vaccines have passed the initial stages of clinical trials [40]. Bivalent EV71 and CVA16 vaccines have also shown efficacy and provided protection against both viruses [18]. However, these vaccines are limited to providing protection only to a fraction of HFMD cases each year. One of the difficulties in developing a broad protecting HFMD vaccine is that enteroviruses are efficient at cross neutralizing between genogroups, but do not cross protect between serotypes [12,16]. Inactivated enterovirus vaccines rely on neutralizing antibodies for protection and the variation between enteroviruses prohibits cross neutralization from occurring. The VP1-VP4 capsid genes (P1 region), are where a majority of neutralizing antibodies bind, but amino acid sequence identity is only 20.7% between CVA16 and EV71, 32.3% between CVA16 and CVA6, and 33.5% between EV71 and CVA6 [41].

Neutralizing epitopes for EV71 and CVA16 have been previously characterized because neutralizing antibodies are the main form of protection against human enteroviruses. Linear epitopes have been shown to map to overlapping areas on the EV71 and CVA16 capsid surface [42]. Crystal structures and VLP models have been solved for both CVA16 and EV71 [42,43,44]. Linear and conformational epitopes for EV71 and CVA16 have been characterized through the use of monoclonal antibodies and synthetic peptide screens. SP55 and SP70 are two linear neutralizing epitopes mapped to the VP1 of EV71 [45]. SP70 is conserved among 25  EV71 sub-genogroups A to C4, while SP55 showed 80% identity with genogroup A, and 85%–100% homology with B1 to C4. Interestingly, SP70 was confirmed as an epitope to EV71 by mapping a monoclonal antibody to the region and conferred 100% protection *in vivo* against EV71 infection [46]. The first conformational neutralization epitope of EV71 was mapped to the knob region of VP3 [47].

Six CVA16 linear neutralizing epitopes have also been determined through screening of synthetic peptides across the VP1 [48]. Amino acid sequences of PEP55, PEP63, and PEP91 are identical among CVA16 strains, PEP32 and PEP37 show 93.3% homology, and PEP71 is conserved among non-A CVA16 genotypes. Neutralizing epitopes for CVA6 have yet to be determined. Groups have recently used this epitope data to make a bivalent chimeric virus-like particle (VLP) vaccines [49,50].

By using a trivalent formulation, we increased protection from HFMD that bivalent and monovalent vaccines cannot. Our trivalent vaccine provided protection to EV71 and CVA16 challenge in mice. We also showed protection to CVA6 by using a passive transfer of serum from trivalent vaccinated mice. Strong neutralizing antibody responses were confirmed before challenge against all three of the viruses included in the trivalent vaccine. However, lower neutralizing antibody levels were detected with the trivalent vaccine when compared to neutralizing antibody levels produced by the monovalent vaccines. Still, the trivalent vaccine provided complete protection against lethal challenge. It is likely that reduction in neutralizing antibody levels resulted from the competition of the immune system to have to build responses to three viruses instead of one. Immune interference has been documented with other trivalent vaccines including human papillomavirus [51]. This vaccine does not include every virus that causes HFMD, but expands protection to the three most prevalent viruses around the world today.

In previous studies we have documented the capability of AG129 mice to produce neutralizing antibodies to viruses within the *Enterovirus A* species from an inactivated vaccine and demonstrated protection from lethal EV71challenge to validate their usefulness for *in vivo* studies [32,33]. We now expand this success to include active vaccinations using inactivated CVA16 with our mouse adapted CVA16 strain; as well as, demonstrate full protection against EV71, CVA16, and CVA6 using an inactivated trivalent vaccine. To our knowledge, this is the first time active vaccination was performed for CVA16 in a 12 week-old mouse model as well as attempting to adapt CVA6 to mice. We were also able to show the lack of cross protection *in vivo* between the enteroviruses by using both homologous and heterologous challenges. This confirms the need and importance of an active challenge model and *in vivo* studies for vaccine efficacy studies.

In conclusion, we have successfully demonstrated the importance of a trivalent vaccine for HFMD by using mouse adapted strains of EV71, CVA16, and CVA6. By adapting EV71 and CVA16 to adult IFN deficient mice we were able to perform active immunization studies as well as explore the immune response of mice to EV71 and CVA16 infection. We still are limited by our CVA6 model, but have improved current mouse susceptibility from neonatal mice to three weeks. The mouse models and vaccines described here will be useful tools to provide a more comprehensive understanding of HFMD, which in turn, will be useful in the development of safe and efficacious vaccines against these pathogens.

## 4. Materials and Methods

### 4.1. Ethics Statement

This study was carried out in strict accordance with recommendations set forth in the National institutes of Health Guide for the Care and Use of Laboratory Animals. All animals and facilities were under the control of the University of Wisconsin-Madison School of Veterinary Medicine with oversight from the UW Research Animal Resource Center. The protocol was approved by the UW Animal Care and Use Committee (Approval #V01458).

### 4.2. Cells and Viruses

Human rhabdomyosarcoma (RD; ATCC# CCL-136) and Vero cells (ATCC# CCL-81) were grown in Dulbecco’s modified minimal essential medium (DMEM; Gibco, Carlsbad, CA, USA) supplemented with 2% fetal bovine serum (FBS), 100 U/mL of penicillin, 100 µg/mL of streptomycin, and incubated at 37 °C with 5% CO_2_. EV71 B2 isolate MS/7423/87 (GenBank: U22522.1) was obtained from Takeda Pharmaceuticals Inc. CVA16 737-Yamagata-1998 (GenBank: AB634323.1) was obtained from Katsumi Mizuta (Yamagata Prefectural Institute of Public Health in Yamagata, Japan), while isolate CVA16 Code: 10057 was received from Steve Oberste (Centers of Disease Control, Atlanta, GA, USA). CVA6 isolate Gdula (GenBank: AY421764.1) was obtained from the Centers of Disease Control (Atlanta, GA, USA). Viral stocks for mouse adaptation were prepared using RD cells following procedures previously described [32]. The EV71, CVA6, and CVA16 (737-Yamagata-1998) were selected for mouse adaptation due to their high viral titers on cell culture. Vaccine preparation for EV71 was prepared using Vero cells as described previously [33]. For CVA6 and CVA16 (10057) vaccine preparation, RD and Vero cells were used. CVA6 RD-infected flasks were frozen at −80 °C after 72 h, whereas CVA16 (10057) infected Vero cells were harvested after 7 days.

### 4.3. Inactivation of EV71, CVA16 and CVA6

EV71 was inactivated using methodology as described previously [33]. A slightly modified protocol was used for inactivation of CVA16 and CVA6. Clarified supernatants were pre-warmed to 37 °C for 1 h prior to inactivation. Ethyleneimine (EI) was added drop-wise to clarified supernatants immediately prior to use. Bottles were sealed and incubated at 37 °C for 7 h with stirring every hour for the first 4 h. Following the incubation, a two-fold excess of sodium thiosulfate was added, stirred for 10 min at room temperature and stored at 4 °C overnight.

Inactivated virus preparations were filtered (0.45 µM) and pelleted by centrifugation at 4 °C, 13,500 rpm for 12 h. Supernatants were removed and pelleted materials were suspended in 10 mL of cold 1× phosphate buffered saline (PBS) overnight at 4 °C. The preparations were vortexed and centrifuged 4 °C, 1200 rpm for 1 min to remove insoluble debris. The supernatants were loaded onto a multistep sucrose gradient and centrifuged 4 °C, 25,000 rpm for 1.5 h (slow acceleration, no braking). Prominent inactivated virus bands for each preparation were combined, washed with cold 1× PBS, and pelleted by centrifugation at 4 °C, 25,000 rpm for 1.5 h. Pellets were suspended in cold 1× PBS, aliquoted and stored at −80 °C.

### 4.4. Characterization of Inactivated CVA6 and CVA16

Inactivation of CVA6 and CVA16 preparations was confirmed via two successive passages in RD cell culture without the observation of cytopathic effect (CPE). The total protein concentration for each inactivated preparation was generated using a BCA assay (Pierce, ThermoFisher, Rockford, IL, USA. Polyclonal mouse serum raised against untreated CVA6 and CVA16 viruses was used for western blot analysis and indirect ELISA to demonstrate that the inactivated viruses retained antigenicity following treatment with EI (data not shown).

### 4.5. Microneutralization Assays

A previously described microneutralization assay was used to measure the antibody response against EV71, CVA16, and CVA6 [32]. RD cells were seeded into 96-well plates at 10^4^ cells per well. When plates were 90% confluent, individual serum samples were heat-inactivated at 56 °C for 30 min. Two-fold serial dilutions of serum samples were mixed with equal volumes of virus suspension at 2,000 TCID_50_/mL and incubated at 37 °C for 1.5 h. 100 µL of each serum-virus mixture was added to three wells (final virus titer 100 TCID_50_ units/well). Each well was scored for CPE at five days post-infection. The end-point neutralizing titer was defined as the highest serum dilution in which at least two of the three replicates were negative for CPE.

### 4.6. Mouse Adaptation of CVA16 and CVA6

Prior to adaptation, it was confirmed that parent (P0) CVA16 and CVA6 were unable to cause clinical signs (weight loss, neurological complications, ruffled fur, limb paralysis, eye irritation) in 4 week-old AG129 mice. Adapted CVA16 (mCVA16) and CVA6 (mCVA6) were produced via sequential passage in mice following a similar strategy described previously [32]. Briefly, one day-old A129 mice (B & K Universal Ltd., Hull, UK) (*n* = 4–7) received intraperitoneal (i.p.) injections of 10^5^ TCID_50_ units in 100 µL of CVA16 (P0). Mice showing clinical signs of infection (neurological signs, weight loss, *etc.*) were euthanized. Brains were aseptically removed and homogenized as 10% suspensions (*w*/*v*) in 1× PBS supplemented with 0.1% bovine serum albumin (BSA). Tissue suspensions were centrifuged at 2500× *g* for 20 min at 4 °C in a Thermo Scientific Sorvall RT1 centrifuge (T41*11210435 rotor). Aliquots of the supernatant were stored at −80 °C and designated as passage one (P1). P1 CVA16 was injected i.p. (100 μL) into groups of 12 day-old A129 mice (*n* = 5). Viral stocks were prepared from the brain, amplified once on RD cells to increase viral titer, and designated as passage two (P2). A third and final passage (P3) was performed by i.p. injections (400 µL) of P2 virus into four week-old AG129 mice (*n* = 5). A 10% brain homogenate from uninfected AG129 mice served as a negative control during passaging experiments. For CVA6 adaptation, five day-old AG129 mice received i.p. injections of 10^7^ TCID_50_ units in 100 µL of the parent virus. 14 day-old and three week-old A129 mice were injected i.p. with 200 µL of P2 and P3 viruses, respectively. Viral stocks of mCVA16 and mCVA6 strains were prepared by a single round of amplification of P3 virus on RD cells. Immediately after euthanasia of mice during final passage, tissue samples of brain, heart, lung, kidney, spleen and intestine were collected from challenged and negative control animals and fixed in 10% neutral buffered formalin. Tissues were paraffin embedded, sectioned and stained with hematoxylin and eosin (H & E). Viral presence in brain homogenates were confirmed via RT-PCR using virus specific primers as previously described [32].

### 4.7. Efficacy of Inactivated HFMD Vaccines

An active immunization was used to test the efficacy of the inactivated HFMD vaccine against EV71 and CVA16 challenge. A passive transfer study was used to test efficacy against CVA6. A 12 week-old mouse model for CVA6 was not achieved through adaptation, preventing active immunization studies. For the active immunization studies, groups of four week-old AG129 mice (*n* = 5–6) were injected intramuscularly (i.m.) with a preparation of 1.5 µg in 100 µL for monovalent vaccines and 4.5 µg in 100 µL (1.5 µg of each antigen) for the trivalent formulation. Alhydrogel 85 (2% final concentration) was added to each vaccine as an adjuvant and continuously rocked at 4 °C for 4 h prior to use. Two negative control groups (*n* = 5–6) received 1× PBS injections (same volume and route). On day 28 post prime, vaccinated mice received an identical booster injection while negative controls received PBS (same volume and route). Serum neutralizing antibody responses against EV71, CVA16, and CVA6 were measured in individual blood samples collected on day 14 post boost (42 post prime). On day 14 post boost, vaccinated and negative control groups were challenged i.p. with either 1.3 × 10^5^ TCID_50_ units in 400 µL of mEV71 or 10^4^ TCID_50_ units in 400 µL of mCVA16. Animals were monitored twice a day for three weeks for clinical signs of disease and mortality.

Passive transfer for CVA6 was done in three week-old A129 mice (*n* = 5–6). A volume of 200 µL was injected i.p. of mouse immune sera raised against the inactivated trivalent vaccine with geometric mean titers (GMT) 640 for EV71, 1280 for CVA16, and 1280 for CVA6. Monovalent CVA6 (GMT = 5120) and a normal mouse serum control groups were also tested (same volume and route). After 24 h, all mice were bled to measure circulating antibodies and challenged i.p. with 2.31 × 10^4^ TCID_50_ units in 400 µL of mCVA6. Mice were monitored and weighed daily for two weeks.

### 4.8. Viral Loads Detected by Real Time RT-PCR

Viral loads were tested in serum samples collected on days 1, 3 and 5 post challenge during active immunization studies and on day 3 for the passive transfer study. SYBR Green dye technology and real-time RT-PCR were used to estimate virus concentration (reported as TCID_50_/mL equivalents). Viral RNA was isolated from 40 µL of serum using a QiaAmp Viral RNA kit (Qiagen, Valencia, CA, USA) and eluted into 40 µL of water. Primer sets targeted a 100 bp region in the VP1 gene (sequences available upon request). Reverse transcription was done using QuantiTect SYBR Green RT-PCR kit (Qiagen, Valencia, CA, USA). All amplifications and fluorescence quantifications were performed using a Bio-Rad iCylcer IQ5 thermal cycler (Bio-Rad, Hercules, CA, USA). A standard curve was used to quantify the total amount of viral RNA in each serum sample. The standard curve was generated from serially diluted samples of viral RNA (mEV71, mCVA16, mCVA6) extracted from known titers of virus (TCID_50_/mL). Cutoff Ct values were determined from negative controls using normal mouse serum. A curve correlation coefficient of 0.995 and a PCR efficiency between 90%–100% was used to validate the assay.

### 4.9. Statistical Analysis

Data from animal studies were compiled in Microsoft Excel and analyzed using GraphPad Prism 6 software (La Jolla, CA, USA). Statistical analysis of weight loss, viral load, and log-transformed reciprocal neutralizing antibody titers were analyzed using a Student’s *t* test, *p*-values reported. Survival curve analysis was performed to assess vaccine effectiveness against EV71, CVA16, and CVA6 challenge using Mantel-Cox test.
